# An investigation on possible effect of leaching fractions physiological responses of hot pepper plants to irrigation water salinity

**DOI:** 10.1186/s12870-019-1910-z

**Published:** 2019-07-08

**Authors:** Rangjian Qiu, Chunwei Liu, Fusheng Li, Zhenchang Wang, Zaiqiang Yang, Ningbo Cui

**Affiliations:** 1 Collaborative Innovation Center on Forecast and Evaluation of Meteorological Disasters, Jiangsu Key Laboratory of Agricultural Meteorology, Nanjing University of Information Science and Technology, Nanjing, 210044 China; 2 College of Agriculture, Guangxi University, Nanning, 530005 Guangxi China; 3 College of Water Conservancy and Hydropower Engineering, Hohai University, Nanjing, 210098 China; 4 State Key Laboratory of Hydraulics and Mountain River Engineering & College of Water Resource and Hydropower, Sichuan University, Chengdu, 610065 China

**Keywords:** Photosynthetic light–response curve, CO_2_–response curve, δ^15^N, Δ^13^C, Photosynthetic capacity

## Abstract

**Background:**

The modification effect of leaching fraction (LF) on the physiological responses of plants to irrigation water salinity (EC_iw_) remains unknown. Here, leaf gas exchange, photosynthetic light–response and CO_2_–response curves, and total carbon (C) and nitrogen (N) accumulation in hot pepper leaves were investigated under three EC_iw_ levels (0.9, 4.7 and 7.0 dS m^− 1^) and two LFs treatments (0.17 and 0.29).

**Results:**

Leaf stomatal conductance was more sensitive to EC_iw_ than the net photosynthesis rate, leading to higher intrinsic water use efficiency (WUE) in higher EC_iw_, whereas the LF did not affect the intrinsic WUE. Carbon isotope discrimination was inhibited by EC_iw_, but was not affected by LF. EC_iw_ reduced the carboxylation efficiency, photosynthetic capacity, photorespiration rate, apparent quantum yield of CO_2_ and irradiance–saturated rate of gross photosynthesis; however, LF did not influence any of these responses. Total C and N accumulation in plants leaves was markedly increased with either decreasing EC_iw_ or increasing LF.

**Conclusions:**

The present study shows that higher EC_iw_ depressed leaf gas exchange, photosynthesis capacity and total C and N accumulation in leaves, but enhanced intrinsic WUE. Somewhat surprisingly, higher LF did not affect the intrinsic WUE but enhanced the total C and N accumulation in leaves.

## Background

In many countries, the shortage of fresh water is a principal factor restricting the development of irrigated agriculture. The use of saline water is a possible alternative to meet the increased water demands for irrigation [1]. A prototypical case is the cultivation of pepper (*Capsicum annuum* L.), which is now one of the most widely grown crops in the world. In 2016, global pepper production (fresh and dry) from some 4 million ha was estimated at some 39 million tonnes, increasing by some 30% in the last decade [2]. Increasing demand for pepper is perhaps not surprising for high nutritional value of pepper. However, the total water requirement for pepper cultivation is by no means small ranging from 500 to 900 mm and up to 1250 mm in some areas [3]. In arid and semi-arid regions where much of the pepper cultivation occurs, fresh water resources are scarce necessitating the use of recycled (and often saline) water. In some areas, up to 1200–1400 mm of saline water with salinity levels ranging from 2.2 to 3.7 dS m^− 1^ have been successfully used to meet pepper water requirements [4]. Unsurprisingly, as with many other crops, irrigation with saline water can result in the accumulation of salt in the root zones, leading to the reduction in pepper growth and yield [5, 6]. Such reduction is the consequence of several physiological responses including lower CO_2_ uptake, intercellular CO_2_ concentration, and availability of intercellular CO_2_ for carboxylation by decreasing stomatal conductance (*g*_*s*_), as well as the reduction in photosynthesis capacity, photosynthesis rate (*P*_*n*_), and depression in both the photochemical and Calvin cycle reactions [7, 8]. To maintain the minimum salinity in the root zones and enhance crop growth, a considerable amount of water is needed to drain salinity when the field is irrigated with saline water [9]. Leaching fraction (LF) is the volume of drainage water passing through the root-zones divided by the volume of irrigation water. Crop yield with saline water irrigation depends on plant evapotranspiration as well as soil salinity leaching [10]. Previous studies have focused on the effects of LF on root growth [11], root-zone salinity, evapotranspiration and yield [10, 12–14]. However, little information is available on the physiological response of hot pepper leaves to LF.

Intrinsic water use efficiency (WUE), defined as the ratio of *P*_*n*_ to *g*_*s*_ at leaf level, can explain instantaneous responses to environmental factors [15]. Intrinsic WUE can be enhanced either by lowering *g*_*s*_, or by maintaining or enhancing the *P*_*n*_ [16, 17]. As salinity stress simultaneously decreases *g*_*s*_ and *P*_*n*_, the intrinsic WUE varies under different salinity levels. Assessing the Brazilian pepper tree (*Schinus terebinthifolius* Raddi), Ewe and Sternberg (2005) [18] reported that the intrinsic WUE did not statistically differ among their salinity treatments, ranging from 0 to 21.4 dS m^− 1^. Likewise, Yarami and Sepaskhah (2015) [19] noted that the intrinsic WUE of saffron (*Crocus sativus*) was not affected when irrigation water salinity (EC_iw_) was lower than 3.0 dS m^− 1^. However, for some crop species, including water melon (*Citrullus lanatus*) [20], henna (*Lawsonia inermis*) [21] and plantain (*Plantago coronopus*) [22], high salinity improved the intrinsic WUE as the sensitivity of *g*_*s*_ to salinity increased relative to *P*_*n*_. Further investigation is therefore necessary to assess whether EC_iw_ and LF can affect intrinsic WUE for hot pepper.

Stable carbon isotope composition (δ^13^C), which is frequently expressed as carbon isotope discrimination (Δ^13^C), has been correlated with gas exchange responses in the plant growth cycle. δ^13^C in plants therefore provides a time–integrated measurement of intrinsic WUE to environmental stress, such as water and salinity stresses [16, 23]. Consequently, the variation of Δ^13^C has been suggested as an indicator of intrinsic WUE since there is a negative relationship between leaf Δ^13^C and intrinsic WUE [15, 24].

Crop nitrogen (N) is important for plant growth. The natural variation of the N isotope composition (δ^15^N) in plants under salinity stress is useful as it is related to N metabolism [23]. Isotope fractionation may occur during the N enzymatic assimilation of nitrate, recycling, translocation, exudation, or volatilization [25, 26]. Salinity–induced impacts on metabolism may cause a substantial change in the isotopic content of metabolites. For instance, increased salinity results in a significant reduction of δ^15^N in wheat shoots, which may result from reduction in the loss of ammonia and nitrous oxide [27]. Many studies have also shown that δ^15^N in plants can be used as an indicator to assess the mineralization rate of soil organic N [28]. Higher δ^15^N in plants indicates more N is absorbed from soil organic N pools than from inorganic mineral N. In addition, the uptake and assimilation of ammonium, plant growth and root length density or surface area may also affect plant N accumulation. Previous studies showed that increasing salinity leads to a reduction in the N content and total N accumulation [23, 27, 29, 30]. However, the modification effect of LF on the uptake of hot pepper N uptake to EC_iw_ remains unclear. In addition, the salinity–induced reduction in hot pepper N may affect C retention in the plant.

Therefore, the objectives of this study are (1) to analysis the response of photosynthetic capacity, intrinsic WUE and total C and N accumulation of hot pepper leaves exposed to different EC_iw_ treatments, and (2) to assess the modification effect of LF on leaf gas exchange, intrinsic WUE, and total C and N accumulation to EC_iw_.

## Results

### Gas exchange, intrinsic WUE, photosynthetic light–response and CO_2_–response curves

**Fig. 1:**
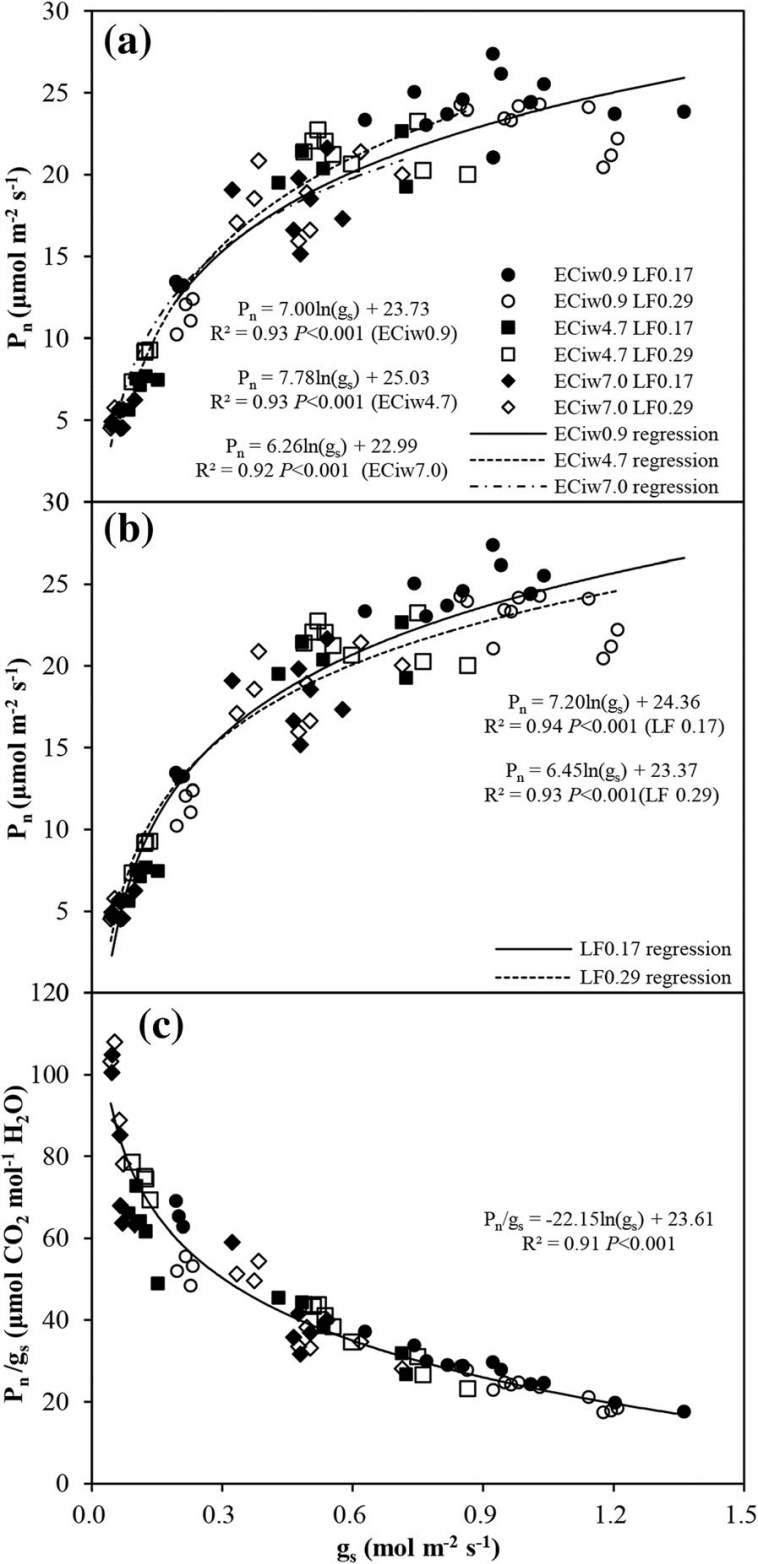
Photosynthesis (*P*_*n*_) and intrinsic water use efficiency (i.e. *P*_*n*_ / *g*_*s*_) (**c**) expressed as a function of stomatal conductance (*g*_*s*_) in the leaves of hot pepper plants under different levels of irrigation water salinity (EC_iw_, **a**) and two leaching fractions (LF, **b**). The data points used were obtained from the pooled data of three measurements of leaf gas exchange (23, 39 and 76 days after transplanting)

Higher EC_iw_ induced the lower *P*_*n*_ and *g*_*s*_. Compared to the EC_iw_ of 0.9 dS m^− 1^, the treatment with EC_iw_ of 7.0 dS m^− 1^ decreased *P*_*n*_ and *g*_*s*_ by 37.7 and 60.5%, respectively, showing that *P*_*n*_ declined slower than *g*_*s*_, which led to a higher intrinsic WUE (i.e. *P*_*n*_ / *g*_*s*_) with higher EC_iw_ (Table 1). Interestingly, high LF did not affect *P*_*n*_ and *g*_*s*_ significantly. As a consequence, the intrinsic WUE had no statistical difference between the two LFs treatments (Table 1). There were significant relationships (i.e., a typical logarithmic correlation) between *P*_*n*_ and *g*_*s*_ under different EC_iw_ levels and LF treatments (Fig. 1a, b), showing that partial stomatal closure would result in an increase in intrinsic WUE [31]. A clear logarithmic decrease of intrinsic WUE with increasing of *g*_*s*_ was also found based on the pooled data from all treatments (Fig. 1c). Collectively, based on these results, it is suggested that EC_iw_ reduced *g*_*s*_ more than *P*_*n*_, resulting in an increase in intrinsic WUE; in contrast LF had no marked effect on *g*_*s*_ and *P*_*n*_, leading to an identical intrinsic WUE. ANCOVA analyses also show that the EC_iw_ × *g*_*s*_ or LF × *g*_*s*_ interactions were not significant, indicating that the slopes of the regression lines between *P*_*n*_ and *g*_*s*_ under different levels of EC_iw_ and LFs were not significantly different. These results also further suggest that at a certain *g*_*s*_, the differences in *P*_*n*_ among the EC_iw_ or LF were consistent (Fig. 1)

**Table 1:** Photosynsthis (*P*_*n*_, μmol m^− 2^ s^− 1^), leaf stomatal conductance (*g*_*s*_, mol m^− 2^ s^− 1^), intercellular to ambient CO_2_ concentration ratio (C_i_ / C_a_) and intrinsic water use efficiency (WUE) (μmol CO_2_ mol^− 1^ H_2_O) in hot pepper leaves subjected to varying levels of irrigation water salinity (EC_iw_, dS m^− 1^) and two leaching fractions (LF). The gas exchange parameters were measured with a fixed PPFD level of 1200 μmol m^− 2^ s^− 1^ (under light saturate condition). The values for each treatment were the averages of three measurements (23, 39 and 76 days after transplanting) with three to six replications for each measurement

Factors	*P*_*n*_	*g*_*s*_	C_i_ / C_a_	Intrinsic WUE
EC_iw_				
0.9	21.2a	0.81a	0.80a	32.6c
4.7	16.0b	0.41b	0.74b	48.8b
7.0	13.2c	0.32c	0.70c	58.4a
LF				
0.17	16.9	0.51	0.75	45.5
0.29	17.5	0.56	0.75	45.4
ANOVA				
LF	NS	NS	NS	NS
EC_iw_	***	***	***	***
LF × EC_iw_	*	NS	*	*

* and *** represent significant differences between means at 0.05 and 0.001 level of probability, respectively; NS, no significant. Different letters within a column indicate significant difference at *P* < 0.05 by Duncan’s multiple range tests

**Fig. 2:**
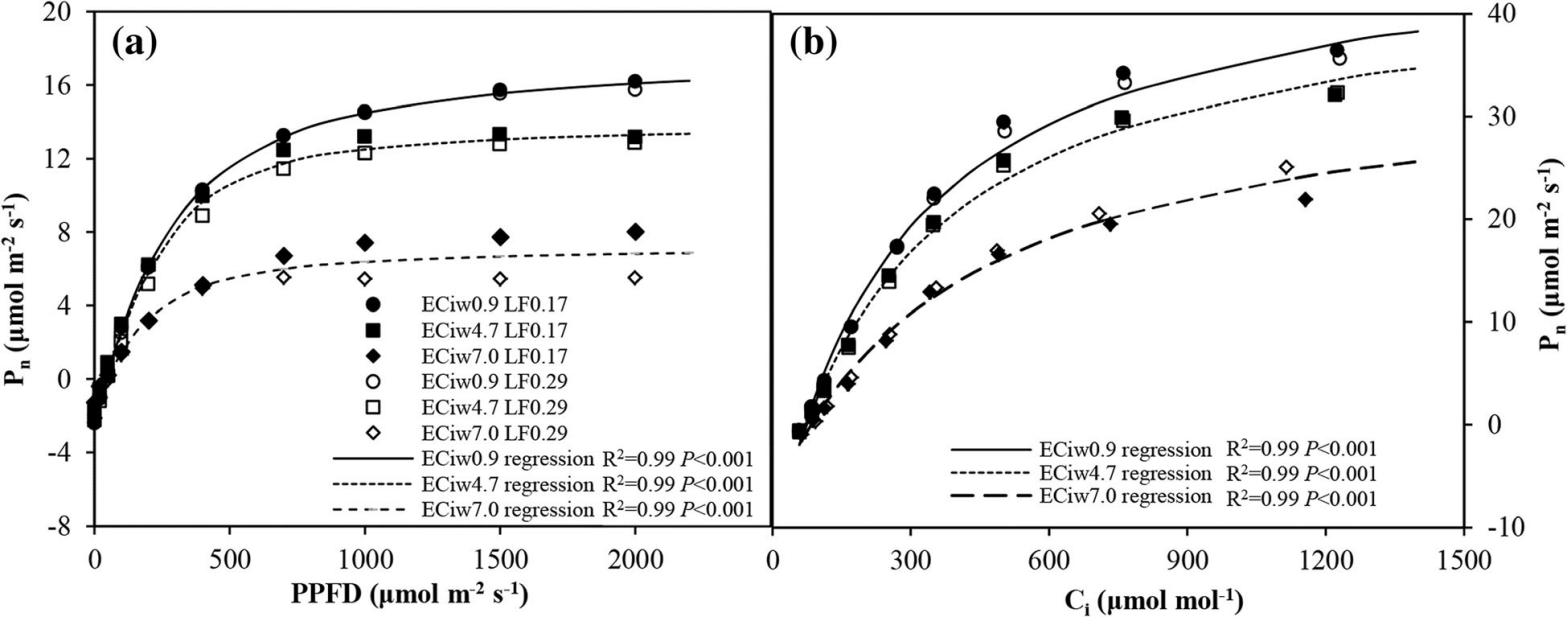
Photosynthetic light–response (**a**) and CO_2_–response curves (**b**) in the leaves of hot pepper plants under different levels of irrigation water salinity (EC_iw_) and leaching fractions (LF) (the measurements were made at a CO_2_ concentration of 400 μmol mol^−1^ and at a PPFD of 1200 μmol mol^− 1^, respectively for light–response curves and CO_2_–response curves). The three regression curves are made for the leaves of EC_iw_ of 0.9, 4.7 and 7 dS m^− 1^, respectively, across the two LFs

The effects of EC_iw_ and LF on gas exchange were further investigated by measuring the photosynthetic light–response (P_n_–PPFD) and CO_2_–response (P_n_–C_i_) curves. Figure 2 shows the P_n_–PPFD and P_n_–C_i_ curves of hot pepper leaves under varying EC_iw_ and LF treatments. The photosynthetic characteristics inculding *α*, *P*_*n max*_, κ and *R*_*d*_ derived from P_n_–PPFD curve and *ε*, *P*_*n sat*_, and *R*_*p*_ derived from P_n_–C_i_ curve are shown in the Table 2. There were no significant interactions between EC_iw_ and LF in terms of the parameters derived from the P_n_–PPFD and P_n_–C_i_ curves. κ was also not influenced by EC_iw_ and LF, indicating *P*_*n*_ increased identically to *P*_*n max*_ as increasing PPFD. The identical *R*_*d*_ under various levels of EC_iw_ and LFs indicate steady early symptom of carbon metabolism [32]. However, salinity–induced reductions in *P*_*n max*_, *α* and *P*_*n sat*_ were observed in this study (Table 2).

**Table 2:** Effects of irrigation water salinity (EC_iw_, dS m^− 1^) and leaching fraction (LF) on maximum apparent quantum yield of CO_2_ (*α*, mol CO_2_ mol^− 1^ photons), irradiance–saturated rate of gross photosynthesis (*P*_*n max*_, μmol m^− 2^ s^− 1^), dark respiration rate (*R*_*d*_, μmol CO_2_ m^− 2^ s^− 1^), and dimensionless convexity term (κ) derived from the photosynthetic light–response curve and on carboxylation efficiency (*ε*, mol m^− 2^ s^− 1^), photosynthetic capacity (*P*_*n sat*_, μmol CO_2_ m^− 2^ s^− 1^), photorespiration rate (*R*_*p*_, μmol CO_2_ m^− 2^ s^− 1^) derived from the photosynthetic CO_2_–response curve. The light–response curves were measured at a fixed CO_2_ concentration of 400 μmol mol^− 1^. Measurements of CO_2_–response curves were conducted at a fixed PPFD of 1200 μmol m^− 2^ s^− 1^

Factors	*α*	*P*_*n max*_	*κ*	*R*_*d*_	*ε*	*P*_*n sat*_	*R*_*p*_
EC_iw_							
0.9	0.052a	19.9a	0.53	2.07	0.224a	61.0a	12.7a
4.7	0.047a	15.7b	0.78	1.78	0.188a	55.7a	11.2a
7.0	0.030b	8.6c	0.83	1.43	0.109b	42.8b	7.5b
LF							
0.17	0.045	16.4	0.64	1.68	0.179	52.1	10.5
0.29	0.042	14.2	0.75	1.96	0.168	54.2	10.4
ANOVA							
LF	NS	NS	NS	NS	NS	NS	NS
EC_iw_	*	***	NS	NS	***	*	**
LF × EC_iw_	NS	NS	NS	NS	NS	NS	NS

*, ** and *** represent significant differences between means at 0.05, 0.01 and 0.001 level of probability, respectively; NS, no significant. Different letters within a column indicate significant difference at *P* < 0.05 by Duncan’s multiple range tests

In agreement with the prior analysis for *P*_*n*_, *g*_*s*_ and intrinsic WUE in this study, the improvement of carboxylation capacity, electron transport, *P*_*n max*_ and *P*_*n sat*_ in the higher LF were not observed on the P_n_–PPFD and P_n_–C_i_ curves (Fig. 2, Table 2), indicating that the higher LF treatment did not enhance *g*_*s*_, which ultimately affected photosynthesis capacity and intrinsic WUE.

### Δ^13^C, δ^15^N and total C and N accumulation in leaves

**Fig. 3:**
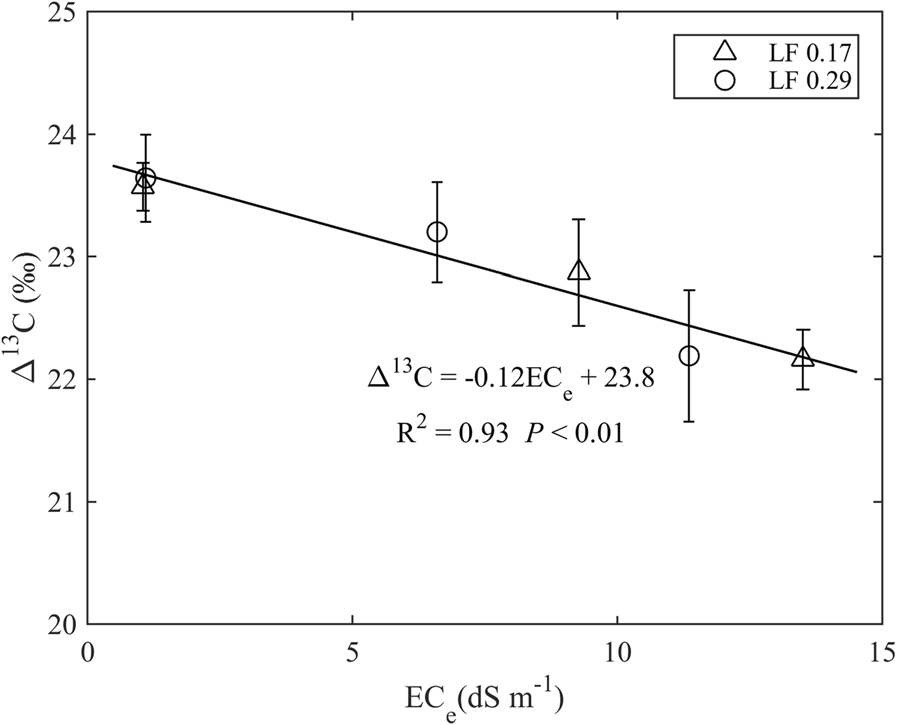
Relationship between carbon isotope discrimination (△^13^C) and electrical conductivity of soil saturated paste extract (EC_e_) regardless of leaching fractions (LF). Values are the means ± SE (*n* = 4)

**Fig. 4:**
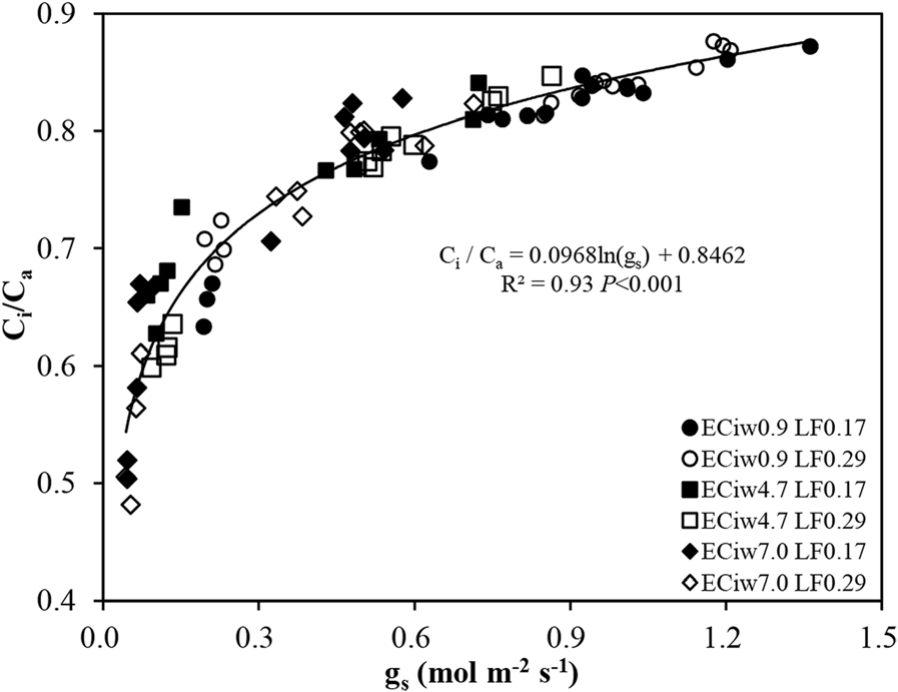
Logarithmic correlation between the intercellular to ambient CO_2_ concentration ratio (C_i_ / C_a_) and stomatal conductance (*g*_*s*_) across the two leaching fractions (LF). The data points used were from the pooled data of three measurements of gas exchange (23, 39 and 76 days after transplanting)

Although no significant interaction between EC_iw_ and LF was found for the Δ^13^C of leaves, Δ^13^C decreased by 2.4 and 6.1% in the EC_iw_ treatments of 4.7 and 7.0 dS m^− 1^, respectively, when compared to the EC_iw_ of 0.9 dS m^− 1^ (Table 3). This suggests that higher EC_iw_ had greater stomatal closure. A significantly negative linear relationship between the Δ^13^C and electrical conductivity of soil saturated paste extract measured at the end of the experiment was observed regardless of the LF treatments (Fig. 3), indicating that soil salinity restricted CO_2_ diffusion in *P*_*n*_ [33]. A previous study has shown that salinity–induced reductions in Δ^13^C accompany decreases in C_i_ / C_a_ [34]. In this study, the decline in Δ^13^C as EC_iw_ increased from 0.9 to 7.0 dS m^− 1^ corresponded to a reduction of C_i_ /C_a_ from 0.8 to 0.7 (Table 1). In addition, a significant positive relationship between the Δ^13^C and C_i_ / C_a_ between the LF treatments was also found (R^2^ = 0.92, *n* = 6, *P* < 0.01). Partial stomatal closure or higher photosynthetic capacity or a combination of both could lead to a decrease in C_i_ / C_a_ [35]. In this study, a significantly positive relationship between C_i_ / C_a_ and *g*_*s*_ represents partial stomatal closure caused by salinity as a result of lower C_i_ / C_a_ levels (Fig. 4, Table 1)

**Table 3:** Carbon isotope discriminaion (Δ^13^C, ‰), C content (% DW), total C accumulation (g plant^− 1^), nitrogen isotope composition (δ^15^N, ‰) and total N accumulation (g plant^− 1^) in hot pepper leaves as affected by varying levels of irrigation water salinity (EC_iw_, dS m^− 1^) and two leaching fractions (LF). The values for each treatment measured at the end of the experiment were the averages of four replications

Factors	Δ^13^C	C content	Total C accumulation	δ^15^N	Total N accumulation
EC_iw_					
0.9	23.61a	40.14a	5.76a	2.44	0.606a
4.7	23.04b	38.54a	3.80b	2.68	0.379b
7.0	22.17c	35.43b	2.36c	2.82	0.238c
LF					
0.17	22.87	36.71b	3.58b	2.69	0.382b
0.29	23.09	39.72a	4.56a	2.58	0.435a
ANOVA					
LF	NS	**	**	NS	*
EC_iw_	***	***	***	NS	***
LF × EC_iw_	NS	NS	NS	NS	NS

*, ** and *** represent significant differences between means at 0.05, 0.01 and 0.001 level of probability, respectively; NS, no significant. Different letters within a column indicate significant difference at *P* < 0.05 by Duncan’s multiple range tests

Previous studies have shown that salinity markedly reduced the δ^15^N in leaves of broccoli and barley plants [36, 37]. However, the δ^15^N in leaves of hot pepper plants was not affect by EC_iw_ (Table 3), indicating that the similar soil organic N mineralization and therefore the identical soil N bioavailability under different levels of EC_iw_ [16]. However, total C and N accumulation in leaves decreased with increasing EC_iw_ (Table 3).

It should be noteworthy that LF did not affect Δ^13^C with values ranging from 22.87 ‰ to 23.09 ‰. Additionally, in accordance with similar Δ^13^C values in two LF treatments, the C_i_ / C_a_ was also identical for two LFs, which may attribute to slimiar stomatal opening and photosynthetic capacity as discussed earlier (Tables 1 and 2). Furthermore, LF also did not influence the δ^15^N in leaves of hot pepper plants. However, higher LF enhanced total C and N accumulation in leaves (Table 3).

## Discussion

Pepper is considerate moderately sensitive to salinity (generally no yield loss when EC_iw_ was lower than 1.5–2.0 dS m^− 1^ [14, 38]). Hence higher EC_iw_ in this study markedly inhibited the *P*_*n*_ and *g*_*s*_, leading to a higher intrinsic WUE. In addition, a significant linear positive correlation between intrinsic WUE and EC_iw_ was observed within the range of EC_iw_ levels considered here regardless of LF treatments (R^2^ = 0.993, *n* = 6, *P* < 0.001). However, additional data on more severe EC_iw_ levels are necessary to assess the aforementioned correlation. For instance, when recalculating data in Table 4 from Chartzoulakis and Klapaki (2000) [6], only a small increase in intrinsic WUE was found when EC_iw_ higher than 12.6 dS m^− 1^, showing that intrinsic WUE did not appreciably increase for the aforementioned correlation.

**Table 4:** Dry biomasses of leaves and roots (g plant^−1^) and Na^+^ content (mg g^−1^ DW) in hot pepper leaves measured at the end of the experiment subjected to varying levels of irrigation water salinity (EC_iw_, dS m^− 1^) and two leaching fractions (LF). Mean values were calculated from four replications

Factors	Dry biomass of leaves	Dry biomass of roots	Na^+^ content
EC_iw_			
0.9	14.3a	5.5a	2.73c
4.7	9.8b	3.4b	8.53b
7.0	7.0c	2.5c	12.60a
LF			
0.17	9.6b	3.6	8.51a
0.29	11.1a	4.1	7.39b
ANOVA			
LF	*	NS	*
EC_iw_	***	***	***
LF × EC_iw_	NS	**	NS

*, ** and *** represent significant differences between means at 0.05, 0.01 and 0.001 level of probability, respectively; NS, no significant. Different letters within a column indicate significant difference at *P* < 0.05 by Duncan’s multiple range tests

Salinity–induced reductions in *P*_*n max*_ and *α* from P_n_–PPFD curves were observed in this study, revealing a comparatively lower capacity of the biochemical reactions responsible for CO_2_ fixation and lower photochemical efficiency of photosystem in hot pepper leaves in higher EC_iw_ [39]. Similarly, *P*_*n sat*_ derived from P_n_–C_i_ curves also restricted in the EC_iw_ of 7.0 dS m^− 1^ treatment as shown by the decline in the initial slope and the level of the upper plateau in the P_n_–C_i_ curve (Fig. 2b) [40]. Brugnoli and Lauteri (1991) [41] observed similar results in bean and cotton plants, with the effect more marked in bean plants. A decline in carboxylation efficiency (*ε*) was a major component among those inhibiting *P*_*n*_ by mesophyll limitations in higher salinity (e.g. EC_iw_ of 7.0 dS m^− 1^ in this study); this was likely produced by a reduction in enzyme activities in the carbon reduction cycle [42]. In addition, owing to the decreases in the CO_2_/O_2_ ratio in the mesophyll, an increase in salinity may increase the rate of photorespiration (*R*_*p*_) in C_3_ plants [8, 43]. However, analysis of the P_n_–C_i_ curves of hot pepper leaves in this study suggested that *R*_*p*_ decreased significantly when EC_iw_ was higher than 4.7 dS m^− 1^ (Table 2). Similar findings have also been reported in mallow [44] and mangrove [45] leaves based on the measurements of gas exchange. The enhanced PEPCase may account for the reduction in *R*_*p*_ [45], however further research is needed to explore the physiological mechanisms of reduced *R*_*p*_ within hot pepper leaves under high salinity levels.

It is well established that Δ^13^C analysis in leaf samples is one of the most versatile methodologies in assessing the environmental effects on the efficiency of photosynthesis in plants [32]. For instance, variation of Δ^13^C was found when plants were subjected to water and salinity stresses [33, 46], which was confirmed by salinity stress in this study. Variation in Δ^13^C relies not only on changes within C_i_ / C_a_, but also the variation in intrinsic WUE [26]. This is confirmed by the negative correlation between the intrinsic WUE and Δ^13^C regardless of LF treatments in this study (R^2^ = 0.92, *n* = 6, *P* < 0.01).

LF did not affect the gas exchange, photosynthesis capacity and hence intrinsic WUE, which further confirmed by the identical value of Δ^13^C. The possible reason is that no creditable soil salinity may leach from root zone in high LF in this study, as indicated by that the electrical conductivites of soil saturated paste extract measured at the end of the experiment were no more than 2.5 dS m^− 1^ between two LFs, especially for lower salinity levels [47].

Higher EC_iw_ induced lower total C accumulation in leaves (Table 3). A lower leaf biomass or a decreased C content in the biomass could retain less C in plant [48]. In this study, lower leaf dry biomass and C content might account for lower total C accumulation in leaves in the higher EC_iw_ treatments (Tables 3 and 4). It is noteworthy that the reduction in leaf dry biomass in higher EC_iw_ levels could result from lower *P*_*n sat*_ and limited root water uptake ability (Table 2). Root water uptake is mainly depended on soil’s matric and osmotic potentials [49, 50]. The salinity reduces the osmotic potential [51], causing the plant to spend more energy in taking up water from the soil solution, leading to a reduction in root water uptake [52, 53]. Salinity–induced reduction of root growth and excessive Na^+^ absorption also limited the root water uptake rate (Table 4).

As expected, high LF enhanced total C accumulation in leaves because of high leaf dry biomass and C content (Table 4), where the enhanced leaf dry biomass in high LF may result from the reduction in Na^+^ uptake and increased osmotic potential (Table 4). However, the reasons for the reduction in C content in higher EC_iw_ and lower LF treatments remain unclear. Wang et al. (2010) [48] suggested that the C content in the plant is affected by the ability of C utilization in the plant. Plant N nutrition is one of the essential factors regulating C metabolism in plants because N is an important element for enzymes concerning metabolism, carbohydrate transport, and utilization in plants [54].

Based on literature surveys, at least four factors may determine plant N uptake from the soil. Firstly, the decreased leaf N accumulation in higher EC_iw_ or lower LF could be attributed to a decrease in plant available N in the soil [28]. If this was the case, the δ^15^N in the high EC_iw_ or low LF treatment should be low because the source of N taken up by plants could be reflected by variations in δ^15^N [55]. However, neither the EC_iw_ nor LF affects δ^15^N in this study (Table 3). Alternatively, the reduced leaf N accumulation may result from the inhibited uptake and assimilation of ammonium as a result of competitive inhibition of Na^+^ [30]. We observed that the Na^+^ content in roots was greater in the higher EC_iw_ and lower LF treatments (Table 4), which might imply that the uptake and assimilation of ammonium was restricted by higher Na^+^ in the higher EC_iw_ and lower LF, and reduced leaf total N accumulation. Thirdly, the reduction in N accumulation in the higher EC_iw_ treatment may result from the decrease in the root surface area for N uptake [28]. Even though the root length density or surface area was not investigated in this study, the root dry biomass declined with increasing EC_iw_ or was not affected by LF (Table 4). This might indicate the lower root density in higher EC_iw_ and similar root density between the two LF treatments. This implies that the lower root length density and root surface area in the higher EC_iw_ might account for the reduction in leaf N accumulation. Lastly, plant N uptake is also affected by plant growth, as shown by significant positive linear correlation between total N content and dry biomass of leaves, regardless of the LFs in this study (R^2^ = 0.98, *n* = 6, *P* < 0.001), indicating leaf total N accumulation was in accordance with the dry biomass accumulation of leaves.

## Conclusions

In summary, our results indicated that higher salinity impacted *g*_*s*_ more than *P*_*n*_, which resulted in higher intrinsic WUE. High salinity also inhibited photosynthesis capacity and retained less C and N in leaves. The novelty of this study is that we found higher LF did not improve leaf gas exchange, photosynthesis capacity and intrinsic WUE. However, higher LF did enhanced C and N accumulation in leaves of hot pepper plants.

## Methods

### Experimental design

The experiment was conducted under a rain shelter from April 28 to July 22, 2015 at the Agro–Meteorology Research Station located in Nanjing, Jiangsu, Eastern China (32.2^°^ N, 118.7^°^ E, altitude 14.4 m). Plastic pots (top diameter 27 cm, bottom diameter 26 cm, and height 22 cm) with holes in the bottom were used. Each pot was filled with 11 kg of air–dried soil (sandy loam, with sand = 75.7%, silt = 20.4% and clay = 3.9%) sieved with a 5–mm sieve. The bulk density of soil was 1.47 g cm^− 3^, field water capacity was 0.27 cm^3^ cm^− 3^ and wilting point was 0.04 cm^3^ cm^− 3^. The electrical conductivity of soil (paste) was 0.59 dS m^− 1^, and the pH was 7.4.

One hot pepper plant (*Capsicum annuum* L., Bocuiwang cultivar, purchased from Jingshiyuan Co. Ltd., China) was transplanted into each pot on April 28, 2015. All the pots were saturated with tap water before the transplanting. Five days after the transplanting, each plant was irrigated using tap water with an irrigation amount of 0.9 L pot^− 1^ (all pots observed drainage). Five days after this irrigation event, three different saline water treatments were initiated for two LFs treatments.

The three EC_iw_ levels assessed were 0.9, 4.7 and 7.0 dS m^− 1^ and the two LFs treatments were 0.17 and 0.29; each treatment was replicated four times. The 24 pots were arranged as a randomized block design. Salinity was increased by adding 1:1 m equivalent concentrations of NaCl and CaCl_2_ to fertilizers (half strength Hoagland solution, see Heeg et al. (2008) [56] and Qiu et al. (2018) [57] for detailed composition). The fertilizers added an electrical conductivity (EC) of 0.9 dS m^− 1^ to the irrigation water for each treatment. The characteristics of the irrigation water for each treatment were shown in Table 5.

**Table 5:** Irrigation water composition used in the experiment. The micro elements of half strength Hoagland solution (in μmol L^−1^: 40 Fe-EDTA, 25 H_3_BO_3_, 2.0 MnCl_2_ × 4H_2_O, 2.0 ZnSO_4_ × 7H_2_O, 0.5 CuSO_4_ × 5H_2_O, 50 KCl, 0.075 (NH_4_)_6_Mo_7_O_24_ × 4H_2_O, 0.15 CoCl_2_ × 6H_2_O) in irrigation water were not shown in table

EC_iw_ (dS m^− 1^)	SAR (mmolc L^− 1^)^0.5^	Cation (mmolc L^− 1^)					Anion (mmolc L^− 1^)			
		Na^+^	Ca^2+^	K^+^	Mg^2+^	NH_4_^+^	Cl^−^	NO_3_^−^	SO_4_^2−^	H_2_PO_4_^−^
0.9	0.0	0	4	2.25	1	0.5	0	6.5	1	0.25
4.7	5.4	17	21	2.25	1	0.5	34	6.5	1	0.25
7.0	7.3	29	33	2.25	1	0.5	58	6.5	1	0.25

The evapotranspiration (ET, g) of each pot was calculated as follows:

1 $$ \mathrm{ET}={\mathrm{W}}_{\mathrm{n}}-\mathrm{W}{}_{\mathrm{n}+1}+\left(\mathrm{AW}-\mathrm{D}\right)\times \uprho $$

where W_n_ and W_n + 1_ are the pot weights before the n^th^ and (n + 1)^th^ irrigation (g); AW and D are the amounts of applied irrigation and drainage water (L), respectively; and ρ is the water bulk density (1000 g L^− 1^).

At each irrigation event, the plants were irrigated with 120 and 140% of ET for each EC_iw_ treatment, which lead to an LF of 0.17 and 0.29 according to the method proposed by Letey et al. (2011) [1]:

2 $$ \frac{\mathrm{AW}}{\mathrm{ET}}=\frac{1}{1-\mathrm{LF}} $$

Therefore a different amount of water based on actual ET for each pot was applied to maintain the target LF. At the end of the experiment, the average actual LF based on the amount of seasonal drainage water and applied water was 0.17 and 0.27, respectively [47], showing that the amount of applied irrigation water is reasonable.

The drainage water of individual pots was collected with a glass bottle positioned beneath each pot, and the amount was collected after each irrigation event. Just before each irrigation event, each pot was weighed with an electronic scale of 20 kg with an accuracy of 0.1 g, afterwards the evapotranspiration and irrigation amounts were calculated. During the experimental period, the plants were irrigated every two to five days and a total of 24 irrigations were applied.

### Leaf gas exchange, δ^13^C and δ^15^N of hot pepper leaves and Na^+^ content in roots

Leaf gas exchange parameters, including *P*_*n*_ and *g*_*s*_, were measured at 9:00–11:00 am on three sunny days (i.e. 23, 39, and 76 days after transplanting) using a portable photosynthesis system with a red–blue light source (LI 6400, LI–COR, Lincoln, NE, USA). Three to six fully grown leaves per treatment were measured with a fixed PPFD level of 1200 μmol m^− 2^ s^− 1^. The intercellular to ambient CO_2_ concentration ratio (C_i_ / C_a_) were also obtained from the gas exchange measurements. As noted earlier, intrinsic WUE is defined as the ratio of *P*_*n*_ to *g*_*s*_.

The plants were harvested on July 22, 2015. The biomasses of the leaves were dried in an oven at 70 °C for 72 h to obtain constant weight. Dry leaf samples were ground and used for δ^13^C and δ^15^N measurements. The values of δ^13^C and δ^15^N as well as the total C and N content in the leaves were measured using a MAT253 Stable Isotope Ratio Mass Spectrometer (Thermo Fisher Scientific, USA). The δ^13^C in leaf dry biomass can be calculated as:

3 $$ {\updelta}^{13}\mathrm{C}=\left(\frac{R_{sample}}{R_{standard}}-1\right)\times 1000 $$

where *R*_*sample*_ and *R*_*standard*_ are the ^13^C/^12^C ratio of the sample and PDB (Pee Dee Belemnite) standard, respectively.

The δ^15^N in the leaf biomass is calculated as:

4 $$ {\updelta}^{15}\mathrm{N}=\left(\frac{R_s}{R_b}-1\right)\times 1000 $$

where *R*_*s*_ and *R*_*b*_ (= 0.3663 at % ^15^N) are the N^15^: (N^14^ + N^15^) ratios of the leaf sample to standard, respectively.

Δ^13^C in leaf dry biomass can be calculated as:

5 $$ {\varDelta}^{13}\mathrm{C}=\frac{\delta {}_a-{\delta}_p}{1+{\delta}_p} $$

where *δ*_*a*_ and *δ*_*p*_ are the carbon isotope composition of source air and plant material, respectively. The *δ*_*a*_ was taken as − 8‰ [34].

The roots of each plant were washed with fresh water, and dried in an oven at 70 °C to obtain constant weight. The dried roots were then ground into a powder, broken down with concentrated HNO_3_ that was warmed with a heating block, and finally dissolved in 5% (v/v) high–purity HNO_3_. The sodium ion (Na^+^) content in the dry roots was determined using an Inductively Coupled Plasma–Optical Emission Spectrometry (ICP–OES, Perkin Elmer Optima 8000). The electrical conductivity of soil saturated paste extract was determined at the end of the experiment by a dual channel pH/mV/Ion/Conductivity benchtop meter (MP522, Shanghai San–Xin Instrumentation Inc., China).

### The P_n_–PPFD and P_n_–C_i_ curves

The P_n_–PPFD and P_n_–C_i_ curves for different levels of EC_iw_ and LFs were determined using a LI–6400 photosynthesis system (LI–COR, Lincoln, NE, USA). The P_n_–PPFD curves were measured at a fixed CO_2_ concentration of 400 μmol mol^− 1^ on 2–4 plants per treatment. Measurements were made at PPFD levels of 2000, 1500, 1000, 700, 400, 200, 100, 50, 20 and 0 μmol m^− 2^ s^− 1^. The non-rectangular hyperbola model was used to simulate P_n_–PPFD curve [58]:

6 $$ {P}_n=\frac{\alpha Q+{P}_{n\kern0.5em \max }-\sqrt{{\left(\alpha Q+{P}_{n\kern0.5em \max}\right)}^2-4\kappa \alpha {QP}_{n\kern0.5em \max }}}{2\kappa }-{R}_d $$

where *P*_*n*_ is the rate of net photosynthesis (μmol CO_2_ m^− 2^ s^− 1^); *Q* is the PPFD (μmol m^− 2^ s^− 1^); *P*_*n max*_ is the irradiance–saturated rate of gross photosynthesis (μmol CO_2_ m^− 2^ s^− 1^); *R*_*d*_ is the dark respiration rate (μmol CO_2_ m^− 2^ s^− 1^) at *Q* = 0; *α* is the maximum apparent quantum yield of CO_2_ (mol CO_2_ mol^− 1^ photons); and κ is a dimensionless convexity term [0, 1].

Measurements of P_n_–C_i_ curves were made at CO_2_ levels of 400, 250, 150, 100, 50, 500, 700, 1000 and 1500 μmol mol^− 1^ at a fixed PPFD of 1200 μmol m^− 2^ s^− 1^. The *P*_*n*_ were plotted against the respective C_i_. A non–rectangular hyperbola curve was used to simulate P_n_–C_i_ curve [59, 60]:

7 $$ {P}_n=\frac{\varepsilon {P}_{n\kern0.5em sat}{C}_i}{\varepsilon {C}_i+{P}_{n\kern0.5em sat}}-{R}_p $$

where *ε* is carboxylation efficiency (mol m^− 2^ s^− 1^); *P*_*n sat*_ is the photosynthetic capacity (μmol CO_2_ m^− 2^ s^− 1^); and *R*_*p*_ is the rate of photorespiration (μmol CO_2_ m^− 2^ s^− 1^).

### Statistic analysis

Two-way analysis of variation using the general linear model-univariate procedure was performed to assess the effects of the EC_iw_ and LF on gas exchange parameters, intrinsic WUE, Δ^13^C, δ^15^N, C content and total C and N accumulation, dry biomass of leaves and roots, Na^+^ content, the parameters obtained from the P_n_–PPFD and P_n_–C_i_ curves. All analyses were conducted in the SPSS software package (Version 21.0, IBM Corp., Armonk, NY). Correlations between the measured parameters were determined with regression analyses. The slopes of the relationships between *P*_*n*_ and *g*_*s*_ under different EC_iw_ levels and LFs were tested by a standard analysis of covariance (ANCOVA). *P*_*n*_ was analyzed through a General Linear Model (GLM) of the natural logarithm of *g*_*s*_. The EC_iw_ (or LF) and the interaction with the linear predictor were included to test for differences in slope. If there was no significant interaction between EC_iw_ (or LF) and linear predictor, the slopes were assumed to be the same.

## Data Availability

The datasets generated and analyzed during the current study are available from the corresponding author on reasonable request. The matlab program fitting P_n_–PPFD and P_n_-C_i_ curves using aforementioned methods in this study were shared freely in https://github.com/shuilibite?tab=repositories.

## Abbreviations

C: Carbon
C_i_ / C_a_: Intercellular to ambient CO_2_ concentration ratio
EC_iw_: Irrigation water salinity
*g*_*s*_: Stomatal conductance
intrinsic WUE: Intrinsic water use efficiency
LF: Leaching fraction
N: Nitrogen
Na^+^: Sodium ion
*P*_*n max*_: Irradiance–saturated rate of gross photosynthesis
*P*_*n sat*_: Photosynthetic capacity
*P*_*n*_: Net photosynthesis rate
P_n_–C_i_: photosynthetic CO_2_–response curve
P_n_–PPFD: Photosynthetic light–response curve
*R*_*d*_: Dark respiration rate
*R*_*p*_: Rate of photorespiration
*α*: Maximum apparent quantum yield of CO_2_
δ^13^C: ^13^C isotope composition
Δ^13^C: Carbon isotope discrimination
δ^15^N: ^15^N isotope composition
*ε*: Carboxylation efficiency
κ: A dimensionless convexity term
